# Hyperspectral Imaging for Predicting the Internal Quality of Kiwifruits Based on Variable Selection Algorithms and Chemometric Models

**DOI:** 10.1038/s41598-017-08509-6

**Published:** 2017-08-10

**Authors:** Hongyan Zhu, Bingquan Chu, Yangyang Fan, Xiaoya Tao, Wenxin Yin, Yong He

**Affiliations:** 1 College of Biosystems Engineering and Food Science, Zhejiang University, Hangzhou, 310058 China; 2 Department of Food Science and Nutrition, Zhejiang Key Laboratory of Agro-Food Processing, Zhejiang University, Hangzhou, 310058 China

## Abstract

We investigated the feasibility and potentiality of determining firmness, soluble solids content (SSC), and pH in kiwifruits using hyperspectral imaging, combined with variable selection methods and calibration models. The images were acquired by a push-broom hyperspectral reflectance imaging system covering two spectral ranges. Weighted regression coefficients (BW), successive projections algorithm (SPA) and genetic algorithm–partial least square (GAPLS) were compared and evaluated for the selection of effective wavelengths. Moreover, multiple linear regression (MLR), partial least squares regression and least squares support vector machine (LS-SVM) were developed to predict quality attributes quantitatively using effective wavelengths. The established models, particularly SPA-MLR, SPA-LS-SVM and GAPLS-LS-SVM, performed well. The SPA-MLR models for firmness (*R*_*pre*_ = 0.9812, RPD = 5.17) and SSC (*R*_*pre*_ = 0.9523, RPD = 3.26) at 380–1023 nm showed excellent performance, whereas GAPLS-LS-SVM was the optimal model at 874–1734 nm for predicting pH (*R*_*pre*_ = 0.9070, RPD = 2.60). Image processing algorithms were developed to transfer the predictive model in every pixel to generate prediction maps that visualize the spatial distribution of firmness and SSC. Hence, the results clearly demonstrated that hyperspectral imaging has the potential as a fast and non-invasive method to predict the quality attributes of kiwifruits.

## Introduction

Fruit quality represents a combination of properties and attributes that determine the suitability of the fruit to be eaten as fresh or stored for a reasonable period without deterioration and confer a value regarding consumer’s satisfaction^1, 2^. Kiwifruit (*Actinidia sp*.) is an emerging horticultural crop globally but is indigenous to the mountains of southern China^3^. Kiwifruit is an economically influential fruit and is appreciated by consumers due to its attractive sensory and nutritional properties, especially its high ascorbic acid content, which is beneficial to health^4, 5^. Currently, kiwifruits are sorted manually or automatically, mainly according to their external characteristics. However, the internal qualities of firmness, soluble solids content (SSC), and acidity may influence the quality evaluation of kiwifruits. Firmness directly impacts the texture, shelf life and consumer acceptance^2^, while SSC and acidity (expressed as pH) determine the flavour of kiwifruits as related to sweetness and sourness, respectively. Moreover, the firmness and SSC are vital parameters in assessing the maturity^6, 7^ for determining the optimal harvest time of kiwifruits.

The conventional methods for quality measurements using standard instrumental methods include a Magness-Taylor penetrometer or texture analyser^8^ for flesh firmness, a digital hand-held pocket refractometer for SSC, and a pH-meter for pH. However, these techniques are destructive, time-consuming or inefficient, involve a considerable amount of manual work and only measure a small percentage of the products^2, 7^. Therefore, non-destructive sensing techniques for assessing the internal quality of kiwifruits are beneficial for ensuring high quality and fast evaluation for consumers, producers, processors, and distributors. Spectroscopic techniques provide the detailed fingerprints of the biological specimen to be analysed for the physical characteristics of the interaction between the electromagnetic radiation and the specimen, such as transmittance, reflectance, absorbance, fluorescence, phosphorescence and radioactive decay^9^. Specifically, visible/near-infrared (Vis/NIR) or near-infrared (NIR) spectroscopy is well established as a non-destructive tool^10^ for multi-constituent quality analysis of fruits, including apples^7^, peaches^2^, citrus^11^, and pears^12^. Furthermore, there has been extensive research regarding the kiwifruit quality assessment based on spectroscopic methods^13–17^. Unfortunately, the inability of NIR spectrometers to capture the internal constituent gradients within fruits may result in discrepancies between the predicted and measured compositions^18^. Furthermore, the spectroscopic assessments with comparatively small point-source measurements do not embody the information on the spatial distribution of quality parameters^18, 19^, which is needed for analysing and monitoring the quality of kiwifruits. Contrastingly, as with hyperspectral imaging, different regions of interest (ROIs) can be selected according to the studies targeted to compensate for the shortcomings of the background noise or point-source measurement.

Originally developed for remote sensing applications, hyperspectral imaging (HSI), which is also known as spectroscopic or chemical imaging, has witnessed tremendous growth and application in diverse fields^20, 21^, specifically in the comprehensive measurements of internal and external quality. HSI has many advantages over the conventional RGB, NIR and multispectral imaging (MSI)^18^. For HSI, there are normally more than 100 bands, while for MSI, there are usually less than 10 bands, which indicates that the spectral information is limited. Moreover, the success of the MSI deeply relies on the efficiency of the HSI for providing the important wavelengths. HSI integrates conventional imaging and spectroscopy to obtain both spatial and spectral information simultaneously from a sample at spatial resolutions varying from the level of single cells up to the macroscopic objects^20, 22^. Hyperspectral images, known as hypercubes, are three-dimensional blocks of data comprising two spatial and one wavelength dimension. As regions of a specimen with similar spectral properties have similar chemical compositions, the hypercube allows for the visualization of the biochemical constituents of a sample, separated into particular areas of the image^18^.

There are most commonly two spectral ranges: 400–1000 nm (Vis/NIR) and 900–1700 nm (NIR), which are encompassed in the HSI system. Extensive studies have been conducted for analysing fruits in the 400–1000 nm range using HSI. These studies include the early detection of apple surface defects/contamination or quality determination (firmness and SSC) in apples^7, 9, 23^; proposing two new indexes of ripening based on three wavelengths close to the chlorophyll absorption peak at 680 nm and comparing the multispectral indexes for the assessment of peach ripening^24^; determining moisture content (MC); firmness and total soluble solids (TSS) of bananas^25^; estimation of MC, TSS, pH in strawberries and texture analysis of the images with grey-level co-occurrence matrix (GLCM)^1^. Contrastingly, a growing body of research has also been conducted to evaluate the fruit quality in the range of 900–1700 nm^26, 27^. Nevertheless, few studies have been generated spatial distribution maps on the quality attributes of kiwifruits using HSI. Moreover, little attention has been paid to determining the better range (400–1000 nm or 900–1700 nm) to predict firmness, SSC and pH based on different variable selection methods and robust calibration models.

Thus, we sought to explore the feasibility and potentiality of determining firmness, SSC, and pH in kiwifruits based on HSI. This utmost goal was achieved by meeting the following specific objectives: (i) establishing a hyperspectral imaging system and choosing a better spectral range for determining the quality attributes by means of full-spectral wavelengths with partial least squares regression (PLSR) model; (ii) comparing and evaluating the superior variable selection method from the weighted regression coefficients (BW), successive projections algorithm (SPA) and genetic algorithm–partial least square (GAPLS) and determining the corresponding optimal wavelengths, which give the highest correlation between the spectral data and the three quality parameters; (iii) developing robust and accurate calibration models [PLSR, multiple linear regression (MLR), least squares support vector machine (LS-SVM)] to quantitatively predict the flesh firmness, SSC, and pH using spectral responses from only the optimal wavelengths; and (iv) applying the optimal model to predict the quality attributes of each pixel in samples and generate spatial distribution maps for the whole kiwifruit.

## Results

### Characteristics of spectral profiles

**Figure 1:**
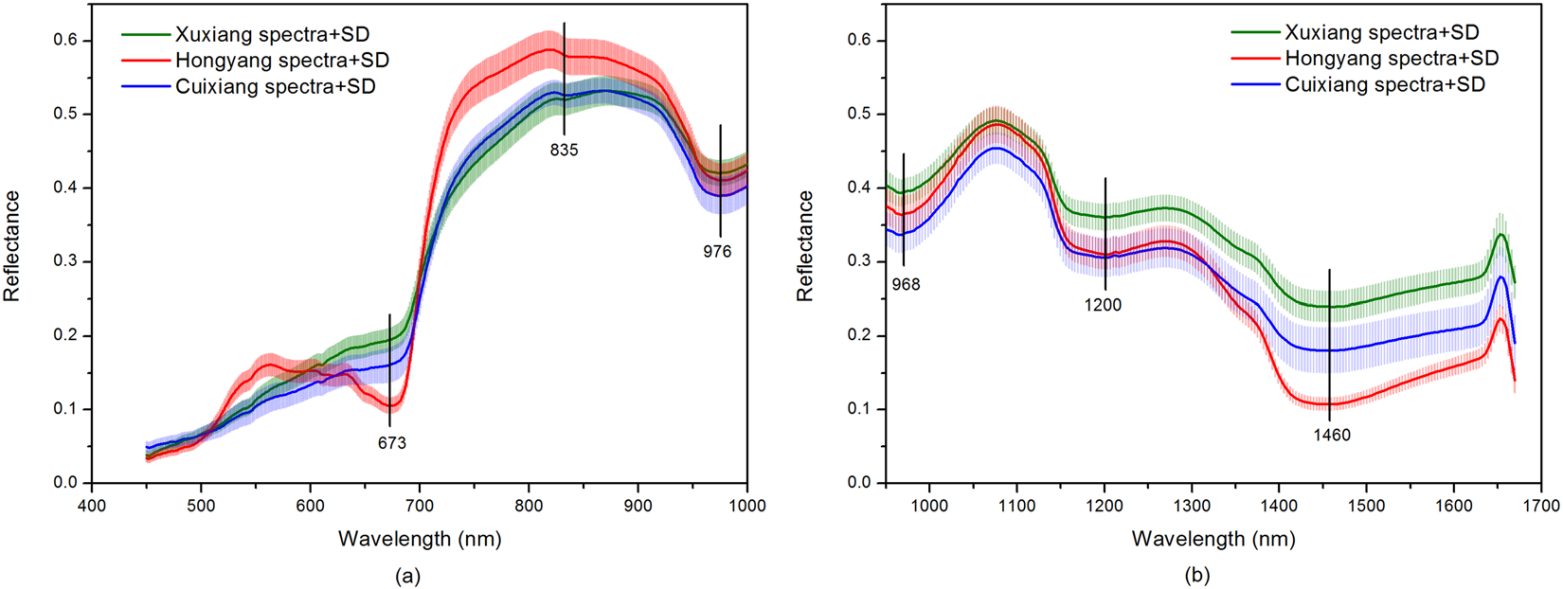
The average reflectance spectra and standard deviation (SD) of ‘Xuxiang’, ‘Hongyang’, and ‘Cuixiang’ at 450–1000 nm (**a**) and 951–1670 nm (**b**).

Figure 1 shows the average reflectance spectra and standard deviation (SD) at the two spectral ranges of ‘Xuxiang’, ‘Hongyang’, and ‘Cuixiang’. There are 512 bands (variables) covering the Vis/NIR spectral range (380–1023 nm). Likewise, the NIR spectral range (874–1734 nm) has 256 bands. However, the spectra in only 450–1000 nm and 951–1670 nm were used for analysis since the beginning and ending of the wavelengths had obviously noisy signals. Substantial absolute variability in the reflectance was observed among the three varieties of kiwifruits between 500 nm and 700 nm, which was mainly caused by different contents of anthocyanin and chlorophyll^2, 28^ (Fig. 1a). Compared with the reflectance spectral region of 450–1000 nm, the average spectrum of each variety at 951–1670 nm (Fig. 1b) showed similar spectral curves and slight differences in reflectance values, with few features. The reflectance curves had four broadband absorption regions of approximately 673, 970 [968 (a), 976 (b)], 1200, and 1460 nm, in addition to a small absorption region at 835 nm.

As shown in Fig. 1a, lower values of reflectance in the visible region were attributed to the dark colour of the kiwifruits due to the relatively homogeneous absorption of light by anthocyanins in the three varieties at 450–550 nm^29, 30^. High absorbance observed at 673 nm indicates red absorbing pigments, particularly chlorophyll, which represents the colour characteristics in the fruit^31^. This result was in agreement with those (675 nm for chlorophyll-*a*) of Cen *et al*.^2^. For the region starting at approximately 700 nm, the reflectance increased rapidly and reached a peak at a wavelength close to 820 nm and another peak at 870 nm. As kiwifruits had an SSC of 10–20% and an estimated water content of 80–90%, the reflectance decreased rapidly from 900 to 1000 nm, which was probably due to the combination effect of O-H groups from the carbohydrates at 835 nm and water at 970 nm. In Fig. 1b, the other two valleys were related to the strong water absorbance bands occurring at 1200 nm and 1460 nm in the kiwifruits^11^. Diverse types of kiwifruits showed different patterns of reflectance spectra, which were characterized by major pigments (chlorophyll-*b*, chlorophyll-*a*, and anthocyanin) and water in the fruit tissue.

### Statistics of measured samples

The descriptive statistics for the quality attributes determined by the standard methods are summarized in Table 1. The tested kiwifruits of each variety were randomly divided into a calibration and a prediction set at the ratio of 2:1. The calibration set consisted of 88 kiwifruits. The prediction set contained 45 kiwifruits used for model validation and verification of the prediction performance of the calibration models. The calculated values varied for the quality attributes of kiwifruits, as illustrated in Table 1. A relatively high variability covering a large scope was anticipated in firmness, which was beneficial in developing a robust calibration model. It may be assumed that the change in storage and transportation of diverse kiwifruit varieties and physicochemical characteristics resulted in the difference in firmness. Nevertheless, the smallest variation was found in the measured pH with a considerably subtle range, which was likely a consequence of the naturally low levels of organic acids in kiwifruits.

**Table 1:** Statistics of quality parameters for 133 kiwifruits measured by standard methods.

Sample Sets	Number	Quality parameters	Range	Mean	SD
Calibration	88	firmness (N cm^−2^)	44.086–642.213	228.559	200.688
		SSC (°Brix)	13.56–18.69	16.02	1.20
		pH	3.64–4.04	3.78	0.09
Prediction	45	firmness (N cm^−2^)	47.756–538.324	220.682	189.78
		SSC (°Brix)	13.09–18.04	15.90	1.33
		pH	3.65–3.81	3.75	0.04

### PLSR models using full spectra

The performances of calibration models are evaluated based on the correlation coefficient (*R*) between the predicted and measured values of attributes, the root mean square error (RMSE) and the residual prediction deviation (RPD). A good model should have a high *R* [calibration (*R*_cal_), cross-validation (*R*_cv_), and prediction (*R*_pre_)], a low RMSE [calibration (RMSEC), cross-validation (RMSECV), and prediction (RMSEP)] and a high RPD^1, 11, 32–34^. In the present study, the PLSR models using the full spectra with a different number of latent variables (LVs) were developed without variable elimination for the prediction of three quality attributes. The PLSR prediction of firmness, SSC, and pH at two spectral ranges are compared and summarized in Table 2. The performances showed that the values of *R*_*pre*_ with various quality parameters varied from 0.6587 to 0.9780 for 450–1000 nm and from 0.8740 to 0.9579 for 951–1670 nm. As shown in Table 2, the overall firmness and SSC had better results in the region of 450–1000 nm, with *R*_*pre*_ = 0.9780 (RPD = 4.71) for the firmness parameter and *R*_*pre*_ = 0.9477 (RPD = 3.12) for SSC, which was consistent with the results obtained in previous studies^11, 35^. Contrastingly, it was observed that the improved results of pH were obtained using the region of 951–1670 nm, with *R*_*pre*_ = 0.8740 (RPD = 2.30). Specifically, when using the better results among two spectral regions, the RPD values all above 2.0 indicated that the PLSR models were robust. We selected a range of 450–1000 nm to predict the firmness and SSC and 951–1670 nm to evaluate the pH for the following analyses.

**Table 2:** PLSR prediction of firmness, SSC, and pH at 450–1000 nm and 951–1670 nm.

Parameter	Spectral range (nm)	Models	LVs	Calibration		Validation		Prediction			
				*R*_*cal*_	RMSEC	*R*_*cv*_	RMSECV	*R*_*pre*_	RMSEP	SEP	RPD
firmness	450–1000	PLSR	4	0.9850	34.3900	0.9828	36.8367	0.9780	39.3580	40.3191	4.71
	951–1670		3	0.9617	54.6751	0.9582	57.1214	0.9579	53.9383	54.4646	3.48
SSC	450–1000		11	0.9510	0.3685	0.9246	0.4548	0.9477	0.4219	0.4260	3.12
	951–1670		12	0.9443	0.3922	0.8806	0.5706	0.9257	0.5607	0.6611	2.01
pH	450–1000		14	0.9259	0.0322	0.8569	0.0441	0.6587	0.0750	0.0935	0.43
	951–1670		4	0.9787	0.0174	0.9765	0.0185	0.8740	0.0178	0.0174	2.30

### Selection of the effective wavelengths

**Figure 2:**
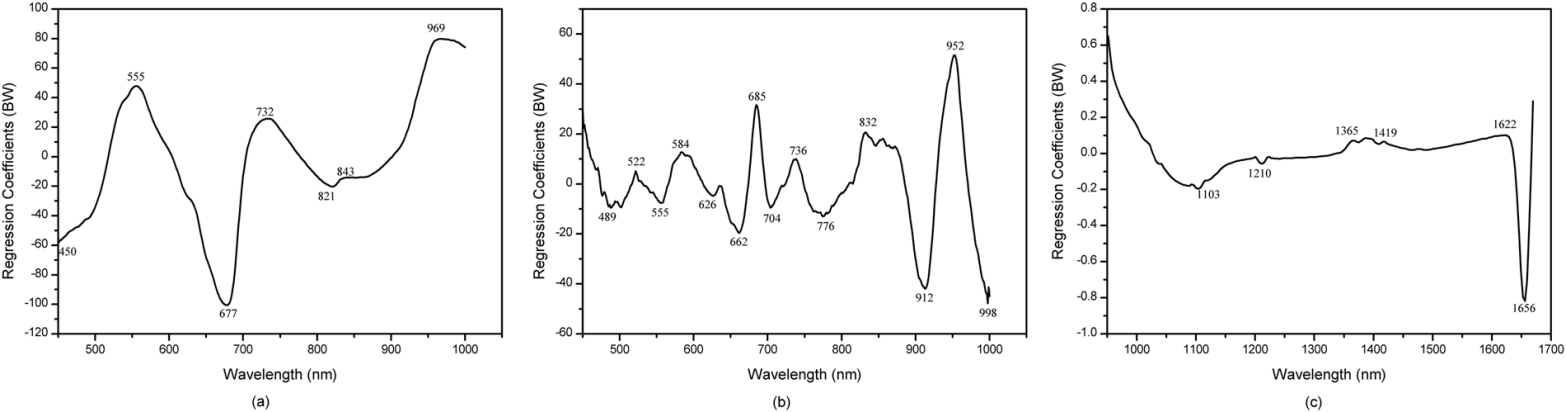
Weighted regression coefficients (BW) resulting from the partial least squares regression (PLSR) models using full spectra for (**a**) firmness, (**b**) SSC and (**c**) pH analysis.

The application with fewer wavebands is preferable for a more stable model and easier implementation in the subsequent multispectral imaging system^36, 37^. Herein, BW, SPA, and GAPLS were used for the selection of effective wavelengths (EWs), which carried the most information for predicting the quality parameters. The EWs selected by the three methods are presented in Table 3. Moreover, BW resulting from the PLSR models were plotted to identify the sensitive wavelengths (Fig. 2). GAPLS and SPA were conducted by programs developed in MATLAB. Overall, GAPLS selected more EWs than the other two methods for quality attributes. Furthermore, the selected EWs by SPA and GAPLS were sequenced in the order of relevance. Different EWs were found to be selected by BW, SPA, and GAPLS due to different variable selection principles. There is no single, universally optimal technique for selecting EWs in a general case. The choice of a selection method bases on the nature of the problem, the size of the dataset, ease of implementation and required accuracy of prediction^20^.

**Table 3:** The selected EWs for quality attributes by BW, SPA and GAPLS.

Parameter	Spectral range (nm)	Methods	No.	Selected EWs (nm)
firmness	450–1000	BW	7	450, 555, 677, 732, 821, 843, 969
		SPA	7	555, 1,000, 638, 503, 452, 700, 822
		GAPLS	8	958, 959, 960, 956, 916, 620, 621, 909
SSC	450–1000	BW	14	489, 522, 555, 584, 626, 662, 685, 704, 736, 776, 832, 912, 952, 998
		SPA	14	987, 960, 487, 496, 737, 814, 450, 945, 912, 643, 558, 522, 451, 692
		GAPLS	27	877, 879, 958, 956, 880, 903, 876, 910, 905, 902, 955, 469, 909, 911, 959, 470, 976, 977, 690, 974, 468, 689, 828, 681, 691, 804, 965
pH	951–1670	BW	6	1103, 1210, 1365, 1419, 1622, 1656
		SPA	5	1659, 1670, 1298, 1133, 999
		GAPLS	12	1670, 1649, 1646, 1653, 1666, 1642, 1656, 1639, 1632, 1636, 1629, 1029

According to the firmness and SSC in the spectral range of 450–1000 nm, the majority of selected EWs by SPA and GAPLS were exhibited in the 600–1000 nm range, which is in accordance with the previous results^11, 29^. Nonetheless, the part of EWs selected by BW were across a range encompassing the visible (450–700 nm) wavelengths. Most previous studies used the short-wave spectral range of NIR (700–1000 nm) to measure the internal fruit quality, which could be attributed to the following two reasons. First, this range is compatible with cheap silicon detectors and is beneficial to light that can penetrate much farther in the fruits of many species^38^. Moreover, this range is relevant to water and sugar since it embodies the second and third overtone of the O-H stretching and vibrations^20^. Correspondingly, a risk of masking spectral information is decreased, although there are low concentrations of constituents in the fruit^11^. Contrastingly, the EWs of pH were exhibited in 1100–1670 nm. The pH values of kiwifruits are likely determined by different kinds of organic acids, such as ascorbic acid, citric acid, malic acid, tartaric acid, and quinic acid. The pH value is related to the organic molecules that contain bonds C–H, O–H, C–O and C–C; hence, it is possible to use NIR methods to determine the pH. Nevertheless, Liu^34^ noted a striking difference in the determination of organic acids of plum vinegar using this window (400–1000 nm) and obtained good results. Although it was possible to select wavelengths corresponding to the known absorption peaks for the different bond groups to quantify chemical compositions of the tested samples, performing this routine for several intrinsic attributes, such as pH and firmness, was impracticable^37^. This is because the NIR spectral region is particularly sensitive to the presence of molecules containing certain functional groups.

### Calibration models and prediction performance

**Figure 3:**
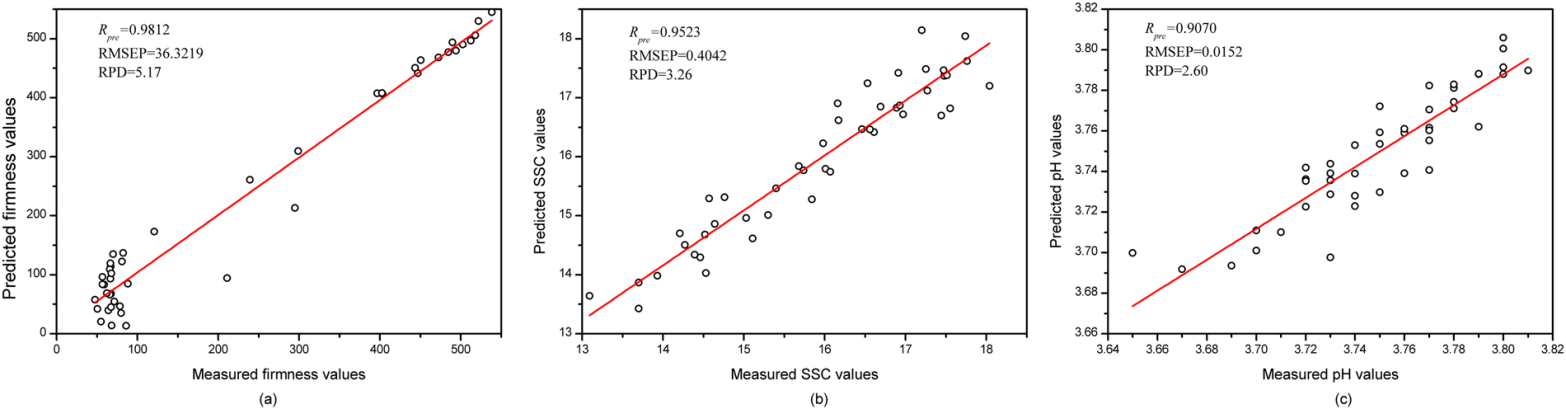
Performances of the best prediction models for detecting quality parameters according to the different effective wavelengths (EWs): (**a**) the combination of successive projections algorithm and multiple linear regression (SPA-MLR) model for firmness, (**b**) the SPA-MLR model for SSC and (**c**) the combination of genetic algorithm–partial least square and least squares support vector machine (GAPLS-LS-SVM) model for pH. Plots represent the actual vs. predicted values of (**a**) firmness, (**b**) SSC, and (**c**) pH.

As a consequence of the previous analyses, different wavelength selection methods prominently reduced the number of wavelengths. The selected EWs were then applied to establish the calibration models instead of the full spectra. To predict the kiwifruit attributes accurately and acquire more information, we applied different chemometric methods and compared them for developing the calibration models. The predictions of firmness, SSC, and pH by the MLR, PLSR and LS-SVM models with the abovementioned EWs are displayed and compared in Table 4. Thus, the newly proposed combination of models were evaluated and compared. The wavelength number was decreased by more than 90% after the wavelength selection by BW, SPA, and GAPLS, which influentially simplified the calibration models and reduced the computation complexity. It can be observed from Table 4 that the performances of firmness, SSC and pH varied among different models. In conclusion, *R*_*cal*_, *R*_*cv*_ and *R*_*pre*_ of all models exceeded 0.88, which indicated that the MLR, PLSR and LS-SVM models performed efficiently. Particularly, Fig. 3 illustrates the performances of the best prediction models for detecting the quality traits according to the different variable selection methods: (a) the SPA-MLR model for firmness, (b) the SPA-MLR model for SSC and (c) the GAPLS-LS-SVM model for pH.

**Table 4:** The results of firmness, SSC, and pH by the MLR, PLSR, LS-SVM models with different EWs.

Parameter	Models	EWs/LVs/(γ, σ^2^)	Calibration		Validation		Prediction			
			*R*_*cal*_	RMSEC	*R*_*cv*_	RMSECV	*R*_*pre*_	RMSEP	SEP	RPD
firmness	BW-MLR	7/−/−	0.9874	31.5520	0.9843	35.2448	0.9711	45.2768	46.7581	4.06
	BW-PLSR	7/3/−	0.9827	36.9834	0.9792	40.5159	0.9746	42.4943	43.9001	4.32
	BW-LS-SVM	7/−/(677.9, 22.5)	0.9983	11.7711	0.9905	27.4618	0.9714	45.9456	47.4716	4.00
	SPA-MLR	7/−/−	0.9848	34.6323	0.9811	38.6382	0.9812	36.3219	36.7222	5.17
	SPA-PLSR	7/3/−	0.9777	41.9495	0.9735	45.6294	0.9646	52.1119	59.1946	3.21
	SPA-LS-SVM	7/−/(266.3, 56.1)	0.9951	19.7982	0.9864	32.8200	0.9821	35.7833	37.4201	5.07
	GAPLS-MLR	8/−/−	0.9883	30.4562	0.9851	34.3028	0.9765	40.7243	41.7817	4.54
	GAPLS-PLSR	8/5/−	0.9873	31.7327	0.9845	34.9907	0.9733	43.2975	43.7160	4.34
	GAPLS-LS-SVM	8/−/(1.1 × 10^7^, 4.2 × 10^3^)	0.9928	23.8324	0.9897	28.5957	0.9695	46.2377	47.1248	4.03
SSC	BW-MLR	14/−/−	0.9282	0.4439	0.8937	0.5376	0.9227	0.5546	0.5131	2.59
	BW-PLSR	14/10/−	0.9237	0.4569	0.8923	0.5423	0.9008	0.6209	0.7567	1.76
	BW-LS-SVM	14/−/(1.0 × 10^7^, 5.5 × 10^4^)	0.9488	0.3799	0.9100	0.4948	0.9183	0.5545	0.6522	2.04
	SPA-MLR	14/−/−	0.9287	0.4423	0.8968	0.5296	0.9523	0.4042	0.4078	3.26
	SPA-PLSR	14/12/−	0.9247	0.4539	0.8948	0.5341	0.9401	0.4645	0.4739	2.81
	SPA-LS-SVM	14/−/(1.2 × 10^7^, 6.4 × 10^4^)	0.9342	0.4295	0.8732	0.5827	0.9485	0.4176	0.4239	3.14
	GAPLS-MLR	27/−/−	0.9547	0.3552	0.9029	0.5186	0.9267	0.5323	0.5052	2.63
	GAPLS-PLSR	27/7/−	0.9332	0.4286	0.9181	0.4730	0.9548	0.4254	0.5161	2.58
	GAPLS-LS-SVM	27/−/(1.5 × 10^7^, 2.4 × 10^4^)	0.9612	0.3300	0.9211	0.4647	0.9437	0.4797	0.4797	2.77
pH	BW-MLR	6/−/−	0.9761	0.0185	0.9722	0.0200	0.8862	0.0187	0.0239	1.67
	BW-PLSR	6/4/−	0.9760	0.0187	0.9722	0.0199	0.8845	0.0184	0.0169	2.37
	BW-LS-SVM	6/−/(5.4 × 10^4^, 824.3)	0.9800	0.0169	0.9731	0.0196	0.8882	0.0181	0.0224	1.79
	SPA-MLR	5/−/−	0.9801	0.0168	0.9777	0.0180	0.8905	0.0162	0.0164	2.44
	SPA-PLSR	5/3/−	0.9786	0.0175	0.9770	0.0185	0.8817	0.0169	0.0171	2.34
	SPA-LS-SVM	5/−/(6.0 × 10^5^, 2.1 × 10^3^)	0.9864	0.0140	0.9825	0.0158	0.9013	0.0157	0.0163	2.45
	GAPLS-MLR	12/−/−	0.9846	0.0152	0.9781	0.0178	0.8977	0.0164	0.0176	2.27
	GAPLS-PLSR	12/3/−	0.9790	0.0173	0.9775	0.0182	0.8814	0.0176	0.0171	2.34
	GAPLS-LS-SVM	12/−/(5.1 × 10^6^, 3.1 × 10^3^)	0.9868	0.0137	0.9820	0.0160	0.9070	0.0152	0.0154	2.60

#### Prediction of firmness

As indicated in Table 4, the MLR, PLSR and LS-SVM models for firmness performed excellently because *R*_*cal*_, *R*_*cv*_ and *R*_*pre*_ of all models were over 0.95. Therefore, it is reasonable to assume that the reference values of firmness covering a large scope were good for developing an accurate and robust calibration model. Briefly, the SPA-PLSR model demonstrated a relatively worse performance than the other models, with a slightly lower RPD (3.21). However, the SPA-MLR and SPA-LS-SVM models performed perfectly, with *R*_*pre*_ (0.9812), RMSEP (36.3219) and *R*_*pre*_ (0.9821), RMSEP (35.7833), respectively. The overall indicator RPD of SPA-MLR (5.17) was higher than that of SPA-LS-SVM (5.07); thus, the SPA-MLR model was deemed the best prediction model for firmness (Fig. 3a). The RPDs of the SPA-MLR and SPA-LS-SVM models were over 5, which revealed that the EWs selected by SPA were more powerful than those of BW and GAPLS.

Notably, the radial basis function (RBF) kernel was recommended as the kernel function of LS-SVM because it could handle the nonlinear relationships between the spectral and target attributes and provide good performance under general smoothness assumptions. Moreover, to achieve an optimal combination of (γ, σ^2^) and avoid overfitting problems, a two-step grid search technique was employed with leave-one-out cross-validation. The ranges of γ and σ^2^ within 10^−2^–10^5^ were set based on the experience and previous research by our team^34, 39^.

#### Prediction of SSC

As illustrated in Table 4, the SPA-MLR and SPA-LS-SVM models in the prediction of SSC with *R*_*pre*_ (0.9523), RMSEP (0.4042) and *R*_*pre*_ (0.9485), RMSEP (0.4176) slightly outperformed the other models, which were parallel to the above analyses of firmness. Apparently, LS-SVM could take advantage of the latent nonlinear information of the spectral data, which contributed to a better prediction performance. Notably, the SPA-MLR model showed the best result (Fig. 3b), which could be related to the procedure of establishing the SPA according to the best MLR model. Contrastingly, the PLSR models yielded slightly poorer results than those of the MLR and LS-SVM models. However, RPDs of all models over 2 demonstrated that the calibration models were robust and accurate, except that the RPD value of 1.76 by BW-PLS was slightly lower for SSC prediction.

#### Prediction of pH

A comparison among Fig. 3a, b and c indicated that the predictions of firmness and SSC performed better than that of pH, which may be attributed to the fact that organic acids concentration in the intact fruits was relatively low^11, 40^. As a result, the calibration of the pH was likely to represent secondary correlations to attributes related to fruit maturity. In Fig. 3c, we present a new combination of the GAPLS-LS-SVM model as the optimal prediction performance for the determination of pH with *R*_*pre*_ (0.9070), RMSEP (0.0152) and RPD (2.60). This indicated that the combination of GAPLS-LS-SVM was constructive and powerful for pH prediction in this specific study, although GAPLS acquired more variables than SPA and BW. Furthermore, the optimal model parameters (γ, σ^2^) in the GAPLS-LS-SVM model were achieved at 5.1 × 10^6^ and 3.1 × 10^3^.

### Spatial distribution maps of firmness and SSC in kiwifruits

The spectral and spatial information of each pixel in the hyperspectral images enabled the evaluation of the quality parameters of each pixel with chemometric models^21, 37, 41^. As the pixels having similar spectral properties would exhibit similar colours in the resulted chemical images, the hypercube allows for the visualization of biochemical constituents of a sample in a pixel-wise manner. However, the measured pH showed the smallest range and variation due to the naturally low levels of organic acids in kiwifruits. Moreover, the pH represented the overall quality of kiwifruit, and the pH of each pixel was meaningless, which might show a similar colour and make it difficult to distinguish based on the pixels of high or low pH. Herein, regarding the firmness or SSC of kiwifruits, the SPA-MLR models obtained the best results with the least EWs, which were best-suited and applied to predict the firmness and SSC of each pixel. The promising quantitative relationship between the spectral reflectance values and the values of firmness or SSC was established through the SPA-MLR models. Two multi-linear functions for firmness and SSC prediction of kiwifruits were obtained:

1 $$\begin{array}{rcl}{Y}_{firmness} & = & -743.0095+5806.358{\lambda }_{{555}nm}+3465.474{\lambda }_{{1000}nm}-3800.496{\lambda }_{{638}nm}\\  &  & -8457.285{\lambda }_{{503}nm}+2374.435{\lambda }_{{452}nm}-425.6517{\lambda }_{{700}nm}\\  &  & -110.9676{\lambda }_{{822}nm}\end{array}$$

2 $$\begin{array}{rcl}{Y}_{SSC} & = & 21.5258-1299.308{X}_{{987}nm}+1163.962{X}_{{960}nm}-870.8445{X}_{{487}nm}\\  &  & +767.1981{X}_{{496}nm}-173.2891{X}_{{737}nm}+209.0592{X}_{{814}nm}+732.1533{X}_{{450}nm}\\  &  & +340.0846{X}_{{945}nm}-299.165{X}_{{912}nm}-60.82231{X}_{{643}nm}+55.44099{X}_{{558}nm}\\  &  & -47.22904{X}_{{522}nm}-502.9426{X}_{{451}nm}+50.60174{X}_{{692}nm}\end{array}$$

where *X*_*i nm*_ is the spectral reflectance value at the wavelength of *i* nm; *Y*_*firmness*_ is the predicted firmness of kiwifruits; *Y*_*SSC*_ is the predicted SSC of kiwifruits.

**Figure 4:**
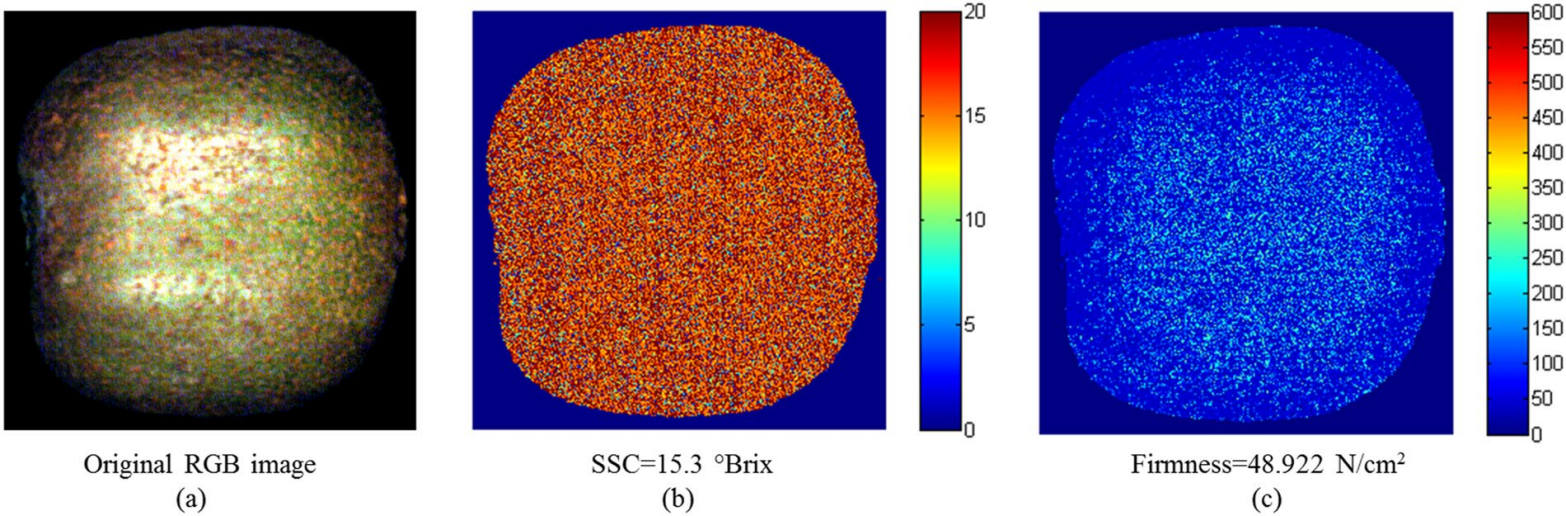
Original RGB image (**a**) and the distribution maps of SSC (**b**) and firmness (**c**) in the kiwifruit (the measured values are on the bottom of the figure). The original RGB image was processed by image calibration and segmentation.

Therefore, the prediction maps of firmness and SSC in the representative kiwifruit generated from the SPA-MLR models were achieved and are spatially presented in Fig. 4. The colour bar in the figure indicates the scale of the values. Pixels providing a similar spectral information in the original hyperspectral images would produce similar colours in the resultant chemical images. Compared with the images of the original samples, the difference in the SSC or firmness and colouring within a sample could be easily identified visually. Figure 4 revealed the changing spatial tendencies of SSC and firmness, which were in alignment with the measured values (SSC = 15.3 °Brix, Firmness = 48.922 N cm^−2^). Remarkably, the noises of the hyperspectral image affected the spectrum of each pixel, which may result in the predicted contents in maps exceeding the range of the calibration set and the prediction set. This issue will be considered by improving the model performances with wider reference value ranges and minimum noises of the spectrum in future work. Overall, the results of the prediction maps indicated that using hyperspectral imaging to estimate the quality parameters of each pixel was feasible.

## Discussion

We have demonstrated the feasibility and usefulness of hyperspectral imaging in the Vis/NIR spectral region (380–1023 nm) and the NIR spectral window (874–1734 nm) for the rapid prediction of quality parameters and mapping the spatial distributions of SSC and firmness in kiwifruits. Three variable selection algorithms, including BW, SPA and GAPLS, were used for EW selection, and different calibration models (MLR, PLSR and LS-SVM) were applied to predict the quality parameters of kiwifruits. This work demonstrated that (1) the spectral profiles had four broadband absorption regions of approximately 673, 970, 1200, and 1460 nm, in addition to the small absorption region at 835 nm, which were mainly due to the pigments, carbohydrates and water in kiwifruits; (2) firmness and SSC were better predicted in the region of 450–1000 nm, and the improved pH results were obtained using the range of 951–1670 nm by means of the full-spectral wavelengths with the PLSR model; (3) SPA and GA-PLS were more powerful than BW, which was the most common method used in previous studies^28, 29^ for the selection of EWs; and (4) the linear and nonlinear calibration models were established for spectral analysis using the EWs. The SPA-MLR models for firmness (*R*_*pre*_ = 0.9812, RPD = 5.17) and SSC (*R*_*pre*_ = 0.9523, RPD = 3.26) in the spectral region of 380–1023 nm showed excellent performance, whereas GAPLS-LS-SVM was the optimal model at the spectral window of 874–1734 nm for predicting pH (*R*_*pre*_ = 0.9070, RPD = 2.60). In addition to predicting the overall average of quality parameters, hyperspectral imaging offers the additional advantage of displaying the distribution of SSC and firmness on the tested samples.

Much pioneering work has been carried out in the kiwifruit quality assessment using spectroscopic methods. McGlone *et al*.^13–15^ demonstrated that NIR spectroscopy was able to predicted firmness, dry-matter and SSC of kiwifruit with PLS models. Additionally, five different wavelength ranges (A, 300–1100; B, 500–1100; C, 500–750; D, 750–1050; E, 800–1000 nm) were also investigated and compared to predict the internal quality parameters of dry-matter, SSC and flesh colour using Vis/NIR spectroscopy^15^. Ragni *et al*.^17^ established an instrumental chain consisting of a sinewave oscillator of waveguide in the microwave field and a frequency analyzer to nondestructively assess the SSC and firmness of “Hayward” kiwifruit. They provided a prediction based on the whole fruit, as opposed to the NIR or the impact mechanical response where the measure is local. Unfortunately, the spectroscopic method, acoustic or mechanical response has a great drawback compared with the hyperspectral imaging because it acquires the spectral data from a single point or from a small portion of the tested fruit. The hyperspectral imaging, on the contrary, has advantages of receiving spatially distributed spectral responses at each pixel of a fruit image. With respect to some of the above mentioned technologies, HSI can obtain spectral signatures at every pixel of an image and offer high resolved spatial spectral analysis, which are significant for distribution maps.

ROI identification, which is a key step in the hyperspectral image processing, should be monitored because the position and size of ROIs have been demonstrated to influence the result^42^. Although no significant difference was observed, this phenomenon indicated the need for careful selection of ROIs in future work. Presently, there are no defined methods or rules that can be used widely to select the ROIs of fruit samples. Evaluating fruit quality by segmenting a small piece of the sample from the whole fruit as ROI has shortcomings. This is because the overall prediction cannot be accurately and reliably achieved by estimating the quality attributes of the ROI, especially when the quality properties of various areas of the fruit are distinctly different^27^. Hence, averaging signals over the whole fruit would be better than using single, small locations for predicting the attributes of fruits. The optimal selection of ROI for this research is the whole fruit region. To augment the generalization, robustness and practicability of the models, it is critical to make some improvements by increasing the number of specimens and investigating wider ranges of the quality attributes. Besides, linear kernel, polynomial kernel, and multilayer perceptron provided alternatives for LS-SVM method instead of just using the RBF kernel. Then the optimal kernel needs to be determined by specific cases. Further work should therefore put emphasis on the optimization of selected EWs, the relationship between the selected EWs and corresponding traits, and the generalization of an expanded model with other similar fruits.

In the light of the present results, it seems feasible to apply HSI as a rapid and accurate alternative to standard texture analysers, traditional digital hand-held pocket refractometers and pH-meters for measuring firmness, SSC and pH, respectively. The practical application of this trend in the fruit industry comes from the fact that visible light combines with the light from the NIR wavelength bands, so the responding system as outlined in this study could be set up for these wavelengths. Once optimized, HSI is anticipated to provide several merits over other traditional techniques in solving the quality control problems since it does not require any consumables or supporting equipment. Extensive studies have proved that different fruit types require differing optimal wavelengths regarding one parameter^1, 25, 43, 44^. Moreover, according to our analysis, it is impossible to use only one MSI system to determine the firmness, SSC and pH simultaneously. Therefore, due to fewer spectral bands and low spectral resolution, the MSI cannot include enough characteristic bands and be comprehensively conducted on the quality attributes of multiple fruit types. Conversely, HSI provides considerably more information about the optical properties of fruits, thus, enabling better prediction of quality parameters in multiple fruit types than MSI^20^. The HSI technique has the potential to detect more quality attributes of fruits, which are less exploited, including colour, bruising, and chemical composition distribution. Thus, additional studies are required before moving the implementation from the near-line application to an on-line approach.

## Materials and Methods

### Sample preparation

Three kiwifruit varieties of ‘Xuxiang’, ‘Hongyang’, and ‘Cuixiang’ were harvested at commercial maturity from Zhouzhi, one of the most famous kiwifruit origins, then immediately transported to the laboratory at Zhejiang University, Hangzhou (120°09′E, 30°14′N), China. Good appearance of the tested fruits was essential. For analysis and image acquisition, we selected 133 wholesome fruits (40, 52 and 41 for ‘Xuxiang’, ‘Hongyang’, and ‘Cuixiang’, respectively) free from any abnormal features such as bruises, diseases, defects, and contaminations. The samples were individually scanned by the hyperspectral imaging system and measured for quality attributes.

### Measurement of quality attributes

Kiwifruit quality attributes were measured after image acquisition. First, flesh firmness was measured at four locations evenly around the equatorial region of each kiwifruit using a texture analyser (TA-XT2i, Stable Microsystems Texture Technologies Inc., UK) fitted with a 5-mm diameter flat probe. The penetration depth was 15 mm, and the test speed was 5 mm/s. Fruit firmness was recorded as Newtons/cm^2^ (N cm^−2^). Then, the kiwifruits were juiced to determine SSC and pH by means of a digital hand-held pocket refractometer (PAL-1, Atago, Itabashi-ku, Japan) and a pH-meter (Sartorius PB-10, Germany) under a temperature of 20 °C. SSC was expressed in °Brix.

### Hyperspectral image collection and processing

#### Hyperspectral imaging system

**Figure 5:**
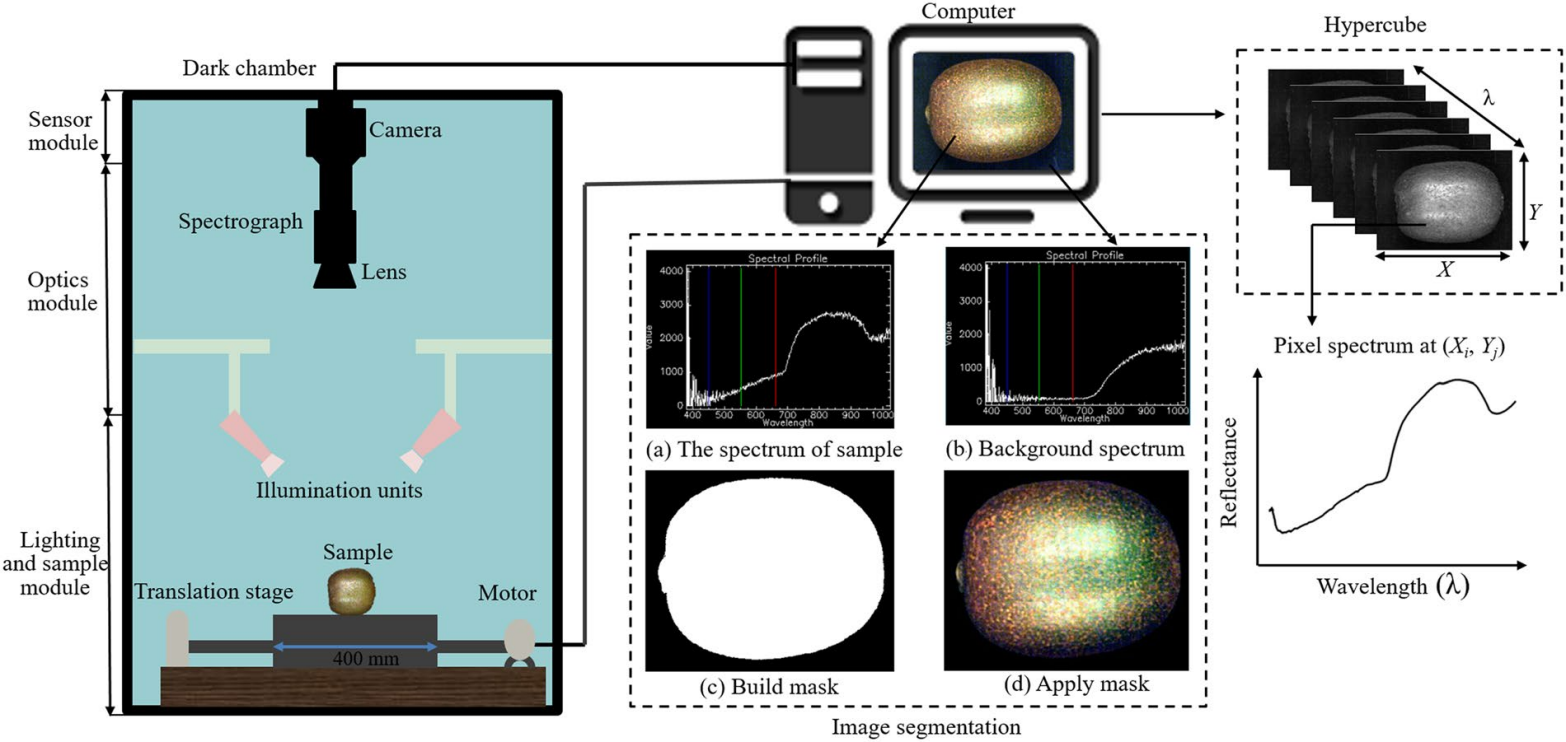
Configuration of the hyperspectral imaging system. The system acquired hyperspectral images at two different spectral ranges (380–1023 nm with 512 bands and 874–1734 nm with 256 bands).

Figure 5 illustrates a typical line-scanning configuration (also called ‘push-broom’) of the hyperspectral reflectance imaging system, which records a whole line of an image rather than a single pixel at a time^37^. The system was placed in a dark room and was composed of the following three modules: (1) A sensor module to control the image acquisition and recording performed with a computer through a data acquisition and system control software (Spectral Image-V10E/N17E, Isuzu Optics Corp, Taiwan, China). (2) The optics module equipped with two imaging spectrographs and cameras, where the Vis/NIR spectral range was acquired by an imaging spectrograph (ImSpector V10E; Spectral Imaging Ltd., Oulu, Finland), a 672 × 512 (spatial × spectral) CCD camera (C8484–05, Hamamatsu, Hamamatsu City, Japan) with a camera lens (OLE23; Specim, Spectral Imaging Ltd., Oulu, Finland). The NIR spectral range was acquired by an imaging spectrograph (ImSpector N17E; Spectral Imaging Ltd., Oulu, Finland), a 320 × 256 CCD camera (Xeva 992; Xenics Infrared Solutions, Leuven, Belgium) with a camera lens (OLES22; Specim, Spectral Imaging Ltd.). OLE23 and OLES22 utilized in this study were the chromatic aberration corrected lens. (3) Lighting and sample module: an illumination unit consisting of two 150 W tungsten halogen lamps (Fibre-Lite DC950 Illuminator; Dolan Jenner Industries Inc, Boxborough, MA, USA) placed on the two sides of the camera symmetrically at an angle of 45° to illuminate the camera’s field of view. The sample was placed on a conveyor belt driven by a stepper motor (Isuzu Optics Corp, Taiwan, China) with an adjustable speed.

#### Image acquisition and calibration

The samples were scanned line by line along the *Y*-axis with the sample moving along the *X*-axis at a certain speed to obtain a three-dimensional hypercube, which encompasses both spatial and spectral information where physical and geometric features and chemical information could be pulled out. To acquire complete, clear and undistorted images, all image acquisition parameters, such as exposure time, height between lens and sample, and motor speed, were adjusted based on the system configuration. For images at 380–1023 nm, the exposure time, the height and the moving speed were set as 0.09 s, 250 mm, and 1.90 mm/s, respectively. For images at 874–1734 nm, the exposure time, height and the moving speed were set as 4 ms, 230 mm and 20 mm/s, respectively. Four kiwifruits were scanned on the conveyor belt with a total length of 400 mm. With a scanning speed of 1.90 mm/s at 380–1023 nm, the time to capture images for each fruit would be 52.63 s $(\frac{400\,mm}{1.90\,mm/s}/4)$. And this time interval would be reduced to only 5 s $(\frac{400\,mm}{20\,mm/s}/4)$ for the case at 874–1734 nm, where the scanning speed was as high as 20 mm/s. Both the two sides around the equator with 180° interval were imaged for each fruit, according to the previous papers^26, 28, 45^. To remove the influence of the dark current of the camera sensor, the calibrated image (*I*) was estimated using the following equation:

3 $$I=\frac{{I}_{raw}-{I}_{dark}}{{I}_{white}-{I}_{dark}}$$

where *I*_*raw*_ was the recorded hyperspectral image, *I*_*dark*_ was the dark reference image (with 0% reflectance) when the light source was turned off and the camera lens was completely covered with its own non-reflective opaque black cap to remove the thermal activities of the CCD detector, and *I*_*white*_ was the white reference image (Teflon white board with 99% reflectance).

### Spectral data extraction

Each time, the system was able to scan four kiwifruits simultaneously, resulting in a hypercube of 672 scanning lines × 1810 spatial pixels × 512 wavelengths between 380 and 1023 nm, with binning operations of 2 × 2 for the spatial and spectral directions, respectively. In contrast, images were binned during acquisition in spatial direction to provide images with spatial dimension of 320 × 858 pixels with 256 spectral bands from 874 to 1734 nm. Segmentation was implemented to segregate each kiwifruit from the background for each hypercube and calculate their mean spectra. The key step was to set a proper threshold and build mask according to the spectra differences between the sample region and the background at a single waveband (700 nm). The ‘build mask’ is a binary image shown in white pixels (1) and the background is shown in black pixels (0). Then we applied mask to the calibrated image after background removal. The masked image with only kiwifruit part is indicated in Fig. 5. The ROI was predefined as the entire fruit region of each kiwifruit to extract spectral data. We used the average reflectance spectrum of all pixels within the ROI to represent the sample. The spectra were obtained from all hyperspectral images of tested kiwifruits and saved in a spectral matrix (*X*).

### Effective wavelengths selection

The spectral and spatial information of all 512 or 256 bands contained redundancy and collinearity, which posed a problem in wavelength selection. EWs selection is very important for spectral analysis^46^. EWs were selected from the original or preprocessed wavelengths and could be directly used for developing the on-line or portable multispectral equipment. Furthermore, EWs could reduce the computation complexity, improve the predictive ability of calibration models, and simplify the calibration models^20^. The wavelengths selection methods used in this research are as follows.

#### BW

BWs are calculated from the best PLSR calibration model were used for selecting the optimal wavelengths^37, 41^. Wavelengths with large absolute values of BW indicate that the variables have influential effects on the prediction of *Y*-variable preference, and can be selected as EWs.

#### GAPLS

GAPLS has been proven to be an effective variable selection method as proposed by Leardi^47^. The basic principle of GAPLS is that the genetic algorithm is implemented by selecting the candidate of sensitive wavelengths and optimizing the number of evaluations in each run, while PLS is applied to perform and evaluate the selected wavelengths. We applied 100 short runs of GAPLS for the variables selection. The sensitive wavelengths selected in each run were recorded, and the weighted frequency of each wavelength selected in 100 runs was calculated. The most frequently selected wavelengths were defined as EWs.

#### SPA

SPA is a feed-forward variable selection method for multivariate calibration, which can greatly reduce the number of variables and improve the speed and efficiency of modelling^34, 46^. To select the wavelength variables with minimum collinearity and redundancy and a maximum projection vector, we conducted the SPA by a simple projection in a vector space. The EWs were finally determined according to the minimum root mean square error of validation (RMSEV) in the validation set of the MLR calibration.

### Chemometric models

#### MLR

MLR establishes a relationship between spectra and the attributes of tested sample in the form of a linear equation with features which is simple and easy to be interpreted^11, 33^. The regression coefficients of this equation are determined by calculating the minimum error between measured and predicted values in a least squares sense. When the number of variables is greater than that of samples, MLR fails and is susceptible to collinearity between variables. Regarding to analyze hyperspectral cubes, the effective wavelength selection or dimensionality reduction is required before MLR model establishment.

#### PLSR

PLSR is a widely utilized multi-analysis and regression method for the quantitative spectral decomposition that is implemented to optimize the covariance between *Y* and the linear combinations of *X* by performing the decomposition on both the spectral and quality data simultaneously^37^. PLSR was carried out to perform the linear models of prediction between the spectral data (*X*-matrix, *N*_samples_ × *K*_wavelengths_) and the values of the parameters obtained from the traditional measurement (*Y*-matrix, *N*_samples_ × 1). The PLSR compresses the spectral data into a set of orthogonal variables called LVs, which carry the most information and maximum covariance between the spectral data and the reference values of the kiwifruit quality attributes. PLSR has been detailed in many previous studies^1, 23, 41^. A full cross-validation (leave-one-out) method was applied to the calibration set to determine the optimal number of LVs.

#### LS-SVM

Increasing evidence from past studies shows that LS-SVM is capable of addressing both linear and nonlinear multivariate analysis problems in a relatively fast way compared with the conventional chemometric methods^2, 34^. Derived from the standard SVM, LS-SVM employs a set of linear equations instead of the quadratic programming problems to obtain the support vectors. Moreover, LS-SVM embodies the structural risk minimization principle to avoid overfitting compared with the traditional empirical risk minimization principle employed by the conventional neural networks. Optimal inputs, a proper kernel function and appropriate LS-SVM parameters were defined before the application of LS-SVM. There were two significant parameters to be decided in an LS-SVM model. The regularization parameter gam (γ) determined the tradeoff between minimizing model complexity and minimizing the training error. The parameter sig^2^ (σ^2^) of RBF kernel function was the bandwidth, which implicitly defined the nonlinear mapping from the input space to some high dimensional feature space^34^.

### Image visualization and distribution map

**Figure 6:**
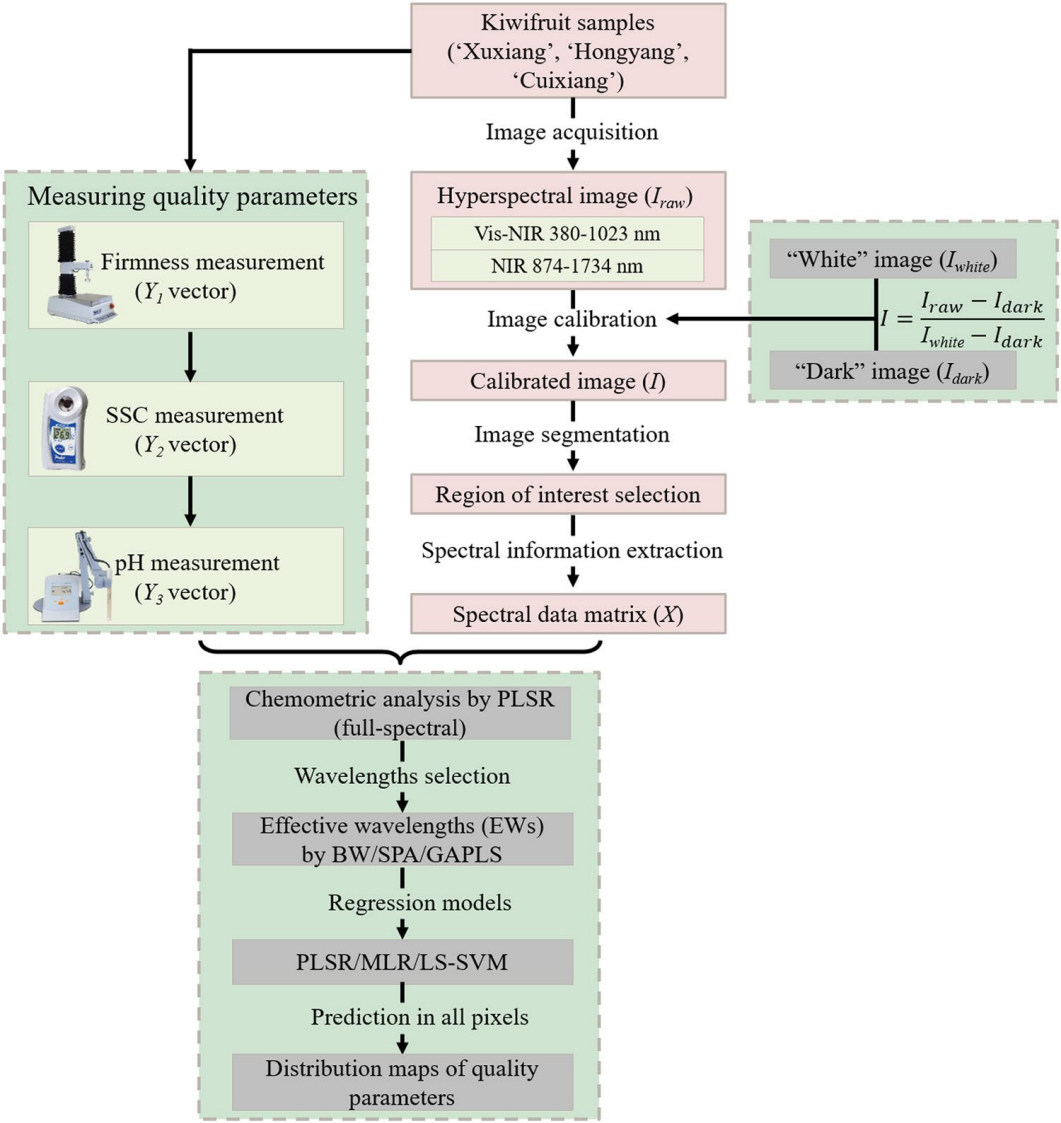
Flowchart of image preprocessing and data analysis for predicting internal quality of kiwifruits.

To observe the difference in kiwifruit quality attributes from sample to sample and even within the same sample, a reduced image space was first formed at the EWs to reduce the amount of time for image analysis. It was practically impossible to obtain the precise quality parameters of every pixel within a sample by chemical analysis, but the quality attributes of every pixel could be predicted by the optimal calibration model. The resulting images could be drawn by using the prediction value of all pixels in the ROI. The results of the distribution map in all spots of the sample facilitate the determination of the difference in the property within one sample as well as among the samples of different sources^37^. Simple, robust, and accurate calibration models are needed to visualize and map the chemical constitution distribution. The key steps for the whole procedure are presented in Fig. 6. Only the distribution maps of SSC and firmness are presented in this study.

### Model evaluation

The statistical parameters mentioned above are defined as follows. Specifically, the RPD values are rated on a scale of 1–6, which indicates the quality of models: 1, inapplicable (RPD < 1); 2, poor (1 ≤ RPD < 1.4) 3, fair (1.4 ≤ RPD < 1.8); 4, good (1.8 ≤ RPD < 2.0); 5, very good (2.0 ≤ RPD < 2.5); 6, excellent (RPD ≥ 2.5)^48^.

4 $$R=\frac{{\sum }_{i=1}^{N}({y}_{i}-\overline{{y}_{i}})({\hat{y}}_{i}-\overline{{\hat{y}}_{i}})}{\sqrt{{\sum }_{i=1}^{N}{({y}_{i}-\overline{{y}_{i}})}^{2}}\sqrt{{\sum }_{i=1}^{N}{({\hat{y}}_{i}-\overline{{\hat{y}}_{i}})}^{2}}}$$

5 $$RMSE=\sqrt{\frac{1}{N}\sum _{i=1}^{N}{({y}_{i}-{\hat{y}}_{i})}^{2}}$$

6 $$RPD=\frac{STD}{SEP}$$

7 $$SEP=\sqrt{\frac{1}{M-1}\sum _{i=1}^{M}{({\hat{y}}_{i}-{y}_{i}-Bias)}^{2}}$$

8 $$Bias=\frac{1}{M}\sum _{i=1}^{M}({\hat{y}}_{i}-{y}_{i})$$

where *y*_*i*_ is the actual value, $\overline{{y}_{i}}$ is the average value of *y*_*i*_, ${\hat{y}}_{i}$ is the predicted value of an attribute in fruit number *i*, $\overline{{\hat{y}}_{i}}$ is the average value of ${\hat{y}}_{i}$, *N* is the number of spectra (samples), and *M* is the number of predicted samples. Spectral data extraction was conducted on ENVI 4.6 (ITT, Visual Information Solutions, Boulder, USA). All computations and multivariate data analyses were performed with the aid of chemometric software Unscrambler® 10.1 (CAMO AS, Oslo, Norway) and MATLAB R 2009b (The Math Works, Natick, USA).
